# Community based telepsychiatry service for older adults residing in a rural and remote region- utilization pattern and satisfaction among stakeholders

**DOI:** 10.1186/s12888-018-1896-3

**Published:** 2018-09-27

**Authors:** Pallavi Dham, Neeraj Gupta, Jacob Alexander, Warwick Black, Tarek Rajji, Elaine Skinner

**Affiliations:** 1 Division of Geriatric Psychiatry, Centre for Addiction and Mental Health, Toronto, Canada; 2 Department of Psychiatry, University of Toronto, Toronto, Canada; 3 Departments of Psychiatry, Rural and Remote Mental Health Services, Adelaide, South Australia; 4 Address on submission: Centre for Addiction and Mental Health, 80 Workman Way, 6th floor, Room 6312, Toronto, ON M6J1H4 Canada

**Keywords:** Telepsychiatry, Elderly, Utilization, Satisfaction, Community

## Abstract

**Background:**

Evaluation of telepsychiatry (via videoconference) for older adults is mostly focussed on nursing homes or inpatients. We evaluated the role of a community based program for older adults in rural and remote regions of South Australia.

**Method:**

The utilization pattern was studied using retrospective chart review of telepsychiatry assessments over 24 months (2010–2011). Satisfaction was evaluated through prospective post-consultation feedback (using a 5-point Likert scale), from patients, community based clinicians and psychiatrist participating in consecutive assessments from April–November 2012. Descriptive analysis was used for the utilization. Mean scores and proportions were calculated for the feedback. Mann Whitney U test was used to compare patient subgroups based on age, gender, prior exposure to telepsychiatry services and inpatient/ outpatient status. Feedback comments were analysed for emerging themes.

**Results:**

On retrospective review of 134 consults, mean age was 75.89 years (SD 7.55), 60.4% (81) were females, and 71.6% (96) lived independently. Patients had a broad range of psychiatric disorders, from mood disorders to delirium and dementia, with co-morbid medical illness in 83.5% (112). On feedback evaluation (*N* = 98), mean scores ranged from 3.88–4.41 for patients, 4.36–4.73 for clinicians and 3.67–4.45 for psychiatrists. Feedback from inpatients (14 out of 37) was significantly lower compared to outpatients (37 out of 61) (chi sq. = 0.808, *p* < 0.05), and they were significantly less satisfied with the wait time (U = 163.0, *p* < 0.05) and visual clarity (U = 160.5, p < 0.05). Audio clarity was the most common aspect of dissatisfaction (mean score less than 3) among patients (6, 11%). Psychiatrists reported a preference for telepsychiatry over face to face in 55.4% (46) assessments. However, they expressed discomfort in situations of cognitive or sensory disabilities in patients.

**Conclusions:**

In rural and remote areas, community-based telepsychiatry program can be a useful adjunct for psychiatrist input in the care of older adults. Innovations to overcome sensory deficits and collaboration with community services should be explored to improve its acceptance among the most vulnerable population.

## Background

Psychiatric disorders among older adults are often complicated by comorbid medical illnesses and disability [1]. Specialist psychiatry input becomes essential in these situations but is often difficult to access, especially among older adults residing in rural and remote regions [2]. One of the key modalities evaluated to meet this need is videoconferencing, also referred to as tele-medicine. It is now a reliable and accepted mode of assessment and treatment in geriatric medicine [3, 4]. In psychiatry, its use is well established among adults and youth in the community setting [5], but among older adults, the evaluation of telepsychiatry is focused on feasibility and diagnostic accuracy in nursing homes and inpatient settings, rather than utilization or satisfaction in the community [6]. It is evaluated to be comparable to face to face evaluation in terms of diagnostic accuracy and is acceptable among the nursing home population [6]. Surprisingly, only one community based telepsychiatry program has been evaluated among older adults [7], utilized mostly by patients with dementia and it showed a high degree of satisfaction among various stakeholders. We lack an understanding about the use of telepsychiatry among older adults for psychiatric disorders other than dementia, especially in the community setting. We do not know if a community based tele-psychiatry program would be widely used, if it would be acceptable to the various stakeholders and what barriers one may face. Evaluation of community-based telepsychiatry programs for this population is essential to get a better understanding of the role of telepsychiatry in mental health service delivery among older adults, especially in rural and remote regions, which can then help guide models of care.

The program we evaluate here differs from the previously evaluated community based telepsychiatry program [7] in its use of high-speed internet at 768 kbps, and a wider coverage of 13 community mental health teams across an area of 980,000 sq. km. It is embedded within an overarching mental health service in the region with a hub of psychiatrists (geriatric psychiatrists; sub-specialty geriatric psychiatry trainee) and mental health nurses in Adelaide. A clinician (nursing or allied health), trained in old age mental health, is embedded in each of the community mental health teams in the rural and remote regions of South Australia and functions as a link between the primary care services in the community and the central hub. Psychiatrist support in assessment and treatment is provided by a combination of community visits by psychiatrists every 4–6 weeks and via telepsychiatry.

The evaluation objective was to describe the pattern of utilization of the telepsychiatry program over 2 years and assess the acceptance and satisfaction among various stakeholders.

## Methods

### Study design

This is a cross-sectional cohort study design with a retrospective chart review component and a prospective feedback survey of stakeholders including the patient, the referring clinician/nurse involved in the patient’s care and the psychiatrist. South Australian Health Ethics committee approved the study.

#### Study sample

- **To assess the pattern of utilization,** the sample included consecutive patients, 65 years and over, with mental health problems, resident in rural and remote South Australia, referred to the service for a tele-psychiatry assessment from January 2010–December 2011.
- **To assess acceptance and satisfaction**, feedback about the tele-psychiatry consultation was sought prospectively from patients, referring nurse/clinician and psychiatrist involved in the tele-psychiatry consultation from April to November 2012. The feedback was voluntary and anonymous. Return of the feedback forms was accepted as consent.

#### Telepsychiatry setup and process of consultation

Telepsychiatry assessment is provided using a secure broadband network at 768 kbps via video conferencing equipment at the Adelaide hub linked to the video-conferencing equipment in community hospitals and health centers in rural and remote regions of South Australia.

Referral from the local General Practitioners (GP) is triaged and a time is booked for an assessment. Referred patient could be residing in the community or nursing home or could be admitted in community hospitals at the time of the assessment. Those residing in the community or nursing homes travel to the nearest community hospital or health center with the videoconferencing equipment. Those admitted to the community hospitals have access to video-conferencing equipment in the hospital premises, but it is not a mobile equipment. A member involved in  the patient’s care (nurse or community mental health team clinician) accompanies the patient, and family members are encouraged to attend. Following an assessment by a psychiatrist, the diagnosis and proposed management plan are conveyed to the patient/family member and the accompanying clinician, and a typed report is sent to the referring GP and the local mental health team. While majority are one time consults, a patient may be reviewed on a repeat request.

#### Data collection

##### To assess the utilization pattern of telepsychiatry services for older patients

We retrospectively gathered information regarding demographics (age, gender, type of residence), physical disability (visual/hearing/physical), Standardised Mini-Mental State Examination (SMMSE) score [8], reason for the referral as mentioned in the referral form, person accompanying the patient (clinician/nurse/family member), comorbid medical illness, psychiatric diagnosis based on the DSM IV criteria and recommendations made, from three sources: client records, telepsychiatry referral form and assessment report sent to the GP. Author PD, using a de-identified standard chart review form approved by the ethics committee collected data on each consultation.

##### To assess acceptance and satisfaction with the service

We prospectively sought post-session feedback from patients, referring clinicians and psychiatrist involved in consecutive telepsychiatry consults from April to November 2012. This was done using standard feedback forms developed for the study and approved by the ethics committee. Telemedicine satisfaction questionnaires have been validated in other areas of medicine [9, 10] and previously used for telepsychiatry among children and adolescents [11] with a primary focus on patient satisfaction. In the absence of validated satisfaction assessment tools in the elderly population, and since we were exploring satisfaction among various stakeholders, we decided to adapt the previously used satisfaction scales to suit the needs of our population and service. The satisfaction questionnaire included questions regarding the referral process, technical aspects, comfort with the use of the medium and satisfaction with the assessment and recommendations (questions detailed in Tables 3 & 5). While the areas covered were similar across the stakeholders, individual questions were modified as relevant to the stakeholders. Each question was rated on a five-point Likert scale with options ranging from “strongly agree” to “strongly disagree”. An open comment section titled “Comments” with 3 lined space allowed provision of additional feedback. The forms were made available to the clinicians and specialists prior to the consult. Accompanying clinician provided the feedback form to the patient at the end of the consult. These were filled in soon after the consult to avoid any recall bias and faxed to the central service after completion. If a patient was unable to provide feedback because of illness severity or cognitive impairment, their significant other (usually a family member) if accompanying the patient, was encouraged to do so.

#### Data analysis

##### Utilization pattern of telepsychiatry services

Descriptive analysis was used to describe the utilization pattern using means with standard deviation (SD) and percentages.

#### Acceptance and satisfaction

Survey respondent and non-respondent patient groups were compared using t test or Pearson’s chi-square Test for any statistical differences in age, gender, living situation, primary psychiatric issue, prior exposure to our telepsychiatry service and inpatient/outpatient status at the time of the assessment.

Responses on each feedback statement were given numerical values from 1 to 5 with 1 representing ‘strongly disagree’ and 5 representing ‘strongly agree’. Mean scores were calculated for each statement. We also calculated the percentage of responses which suggested satisfaction i.e. ≥4, (‘agree’ or ‘strongly agree’) or dissatisfaction i.e. ≤2 (‘disagree’ or ‘strongly disagree’) on each statement. Mann-Whitney Rank Sum Test was used to compare feedback scores on each question based on gender, previous experience with telepsychiatry, independent living/nursing home and inpatient/outpatient status. All analyses were performed using SPSS version 23.0.0.0.

We manually analyzed feedback comments received from each group to look for emerging themes. This was done by NG and verified by PD. The comments were first classified as positive (if the feedback was favorable) or negative (if the feedback was unfavorable). The content of the individual comments was categorized into sub-categories or themes, such that one or more sub-category might apply to each comment. Three sub-categories were identified: a) corresponding to the items covered in the feedback form; b) global themes not linked to a specific item on the feedback form; and c) other statements which could not be reconciled with the feedback form items or global themes. They were then expressed as counts (Table 6) and selected verbatim quotes are mentioned in the results section.

## Results

### Profile of older adult seen via telepsychiatry in 2010–2011

In 2010–11, 140 telepsychiatry consults were performed, of which we analyzed the data from 134 consults of 101 patients, averaging 1.32 consult per patient. The missing data relates to deceased patients whose files were not accessible.

The demographic details and clinical profile of the patients seen via telepsychiatry are described in Table 1**. Their** mean age was 75.89 years (SD 7.55), majority were females (81, 60.4%) and most lived independently (96, 71.6%). Up to 90 patients (67.2%) had no documented disability (defined as visual/hearing/physical/speech). Mean SMMSE [8] score was 24.4 (SD 4.98). Majority had two or more medical conditions (96, 71.6%), including hypertension, diabetes, infections, hypothyroidism, renal failure, cardiac disease, cerebrovascular accidents, seizure, pain, and cancer.

**Table 1:** Profile of patients seen via tele-psychiatry from January 2010 to 2011

Consumer profile	Jan 2010 to Dec 2011 (*N* = 134)
Mean Age (SD)	75.89 (SD 7.55)
Female (n, %)	81 (60.4%)
Place of residence (n, %)	
Private own/rental, Independent house	96 (71.6%)
Residential Age Care Facility (RACF)	25 (18.7%)
Hospital	7 (5.2%)
Not stated	6 (4.5%)
Physical disability (n, %)	
No disability	90 (67.2%)
Visual/hearing impaired	21 (15.7%)
Unsteady gait/ restricted mobility	17 (12.7%)
Not stated	6 (4.5%)
Difficult Speech/aphasia	2 (1.5%)
Medical Illnesses (eg: hypertension, diabetes, infections, renal impairment, cancers) (n, %)	
2 or more medical conditions	96 (71.6%)
Single medical condition	16 (11.9%)
No medical illness	17 (12.7%)
Not stated	5 (3.7%)
Standard Mini Mental State Examination (SMMSE)	
Mean score (SD)	24.44 (SD 4.98)
Not mentioned (n, %)	61 (45.5%)
Reason for referral (n, %)	
Depression/anxiety	58 (43.3%)
Difficult-behaviors (odd/confused/paranoid/manic/aggression/uncooperative)	26 (19.4%)
Not specified	28 (20.9%)
Self-harm ideation/attempt	16 (11.9%)
Others (alcohol use, medication side effects, capacity)	6 (4.5%)
Person accompanying the patient (n, %)	
Member of the Community Mental Health Team (CMHT) /clinician	69 (51.5%)
Clinical nurse (RACF/hospital/practice)	56 (41.8%)
Family (spouse/son/daughter)	53 (39.6%)
Not stated	9 (6.3%)
GP	5 (3.7%)
DSM IV diagnosis (n, %)	
Major Depressive Disorder	51 (38.1%)
Bipolar Disorder	20 (14.9%)
Dementia	18 (13.4%)
Schizophrenia/Schizo-affective disorder/Delusional Disorder/Psychotic Disorder NOS	14 (10.5%)
Anxiety disorders (PTSD, Panic Disorder, NOS)	11 (8.2%)
Adjustment Disorder	11 (8.2%)
Delirium	9 (6.7%)
Medication side effects	7 (5.2%)
No axis 1 diagnosis	3 (2.2%)
Substance dependence/abuse	2 (1.5%)
Recommendations provided (n, %)	
Medication recommendation only	55 (41.0%)
Medication recommendation + psychosocial supports and services	32 (23.9%)
Recommended further medical evaluation/inpatient admission+/− medication recommendation	34 (25.4%)
No further changes	13 (9.7%)

Depression and anxiety were the most common reasons for referral to the telepsychiatry service (58, 43.3%). Difficult behaviors (odd, confused, paranoid, aggressive or uncooperative) and self-harm formed a smaller percentage of the referrals (26, 19.4% and 16, 11.9% respectively, Table 1). Major Depressive Disorder was the most common psychiatric diagnosis (51, 38.1%), although the list of psychiatric disorders was diverse including adjustment disorder, bipolar disorder, psychotic disorder, dementia and delirium **(**Table 1**)**. A clinician from the community team accompanied 69 (51.5%) patients and a family member accompanied 53 (39.6%) consults. In 87 consults (64.9%), recommendations included medication changes with or without other psychosocial assessment/ treatments and 34 (25.4%) patients were recommended inpatient treatment or further medical evaluation.

### Prospective feedback from patients, clinicians, and psychiatrists for telepsychiatry consultations

The sample population providing the feedback was very similar in terms of mean age (75.89, SD 7.55 and 75.68, SD 6.39) and gender distribution (60–65% females) to the sample used for evaluation of the utilization pattern. Further, depression and anxiety disorders were the most prevalent diagnosis in both populations and severe mental illness, dementia and delirium had a lower prevalence (Tables 1 and 2). The details of the feedback are described below.

**Table 2:** Profile of patients seen via tele-psychiatry seen during the period of feedback review in 2012

	Total (N=98)	Feedback received (*N* = 51)	Feedback not received (*N* = 47)	Statistical significance
Mean age in years (SD)	75.68 (6.39)	75.94 (6.45)	75.40 (6.39)	*t* = 0.414, *p* = 0.860^a^
Gender				
Females (N, %)	64 (65.3%	34 (66.7%)	30 (63.8%)	χ^2^ = 0.087 *p* = 0.833^b^
Males (N,%)	34 (34.7%)	17 (33.3%)	17 (36.2%)	
Prior tele-psychiatry contact with the service (N, %)				χ^2^ = 1.791 *p* = 0.216^b^
Yes	38 (38.8%)	23 (45.1%)	15 (31.9%)	
No	60 (61.2%)	28 (54.9%)	32 (68.1%)	
Primary Psychiatry Diagnosis made at the assessment (N, %)				
Depression or anxiety	51 (52.0%)	30 (58.8%)	21 (44.7%)	χ^2^ = 9.107 *p* = 0.168^b^
Mania	10 (10.2%)	5 (9.8%)	5 (10.6%)	
Psychotic illness	21 (21.4%)	12 (23.5%)	9 (19.1%)	
Delirium	6 (6.1%)	1 (2.0%)	5 (10.6%)	
Organic mood disorder	2 (2.0%)	1 (2.0%)	1 (2.1%)	
Dementia	7 (7.1%)	1 (2.0%)	6 (12.8%)	
Unclear	1 (1%)	1 (2.0%)	0 (0.0%)	
Living Situation (N, %)				
Independent living (Home or Retirement home)	82 (83.7%)	41 (80.4%)	41 (87.2%)	χ^2^ = 1.229 *p* = 0.420^b^
Nursing Home	16 (16.3%)	10 (19.6%)	6 (12.8%)	
Status at consultation (N, %)				
Inpatient (admitted in community hospital)	37 (37.8%)	14 (27.5%)	23 (48.9%)	χ^2^ = 0.838 p = 0.037* ^***b***^
Outpatient	61 (62.2%)	37 (72.5%)	24 (51.1%)	

^a^ = t test; ^b^ = Chi Square test, *significance at *p*< 0.05

#### Feedback from patients

Table 2 describes the profile of patients who participated in the feedback survey between April to November 2012 (*N* = 98). Their mean age was 75.68 years (SD 6.39) and 64 (65.5%) were females. Majority were new consults (60, 61.2%). Feedback was received from 51 patients (52%), of which family members provided the feedback in 5 consults. The respondents and non-respondent patient groups did not differ statistically based on mean age, gender, previous contact with the service, primary psychiatric diagnosis or independent living versus residence in a nursing home. The feedback however was significantly lower among patients admitted in community hospitals (inpatients) compared to outpatients (χ ^2^ = 0.808 *p* = 0.037, Table 2). Feedback rates were also low in dementia and delirium (provided by significant other), but the difference based on diagnosis was not statistically significant **(**Table 2**).**

Table 3 outlines the mean scores on feedback ranging  between 3.88–4.41, mean scores greater than 4 on ten out of twelve feedback questions across the domains of process of referral and wait times, technical aspects, comfort and satisfaction with the consultation and recommendations. Satisfaction with the consultation was high with a mean score of 4.14 ± 0.75. While overall expression of dissatisfaction was minimal (2% to 11.8%), most common dissatisfaction was on question 5 i.e. “I was able to hear clearly” **(**6, 11.8%, Table 3). On most questions they were decisive regarding their satisfaction or dissatisfaction, but a few were unsure if the recommendations were useful on question 9 (12, 23.5%) or if their needs were met on question 8 (14, 27.5%).

**Table 3:** Feedback scores for patients seen via tele-psychiatry in 2012 (N = 51)

Feedback Question	Mean ± SD	Percentage satisfied^a^ (n)	Percentage unsure^a^ (n)	Percentage dissatisfied^a^ (n)
1. The waiting period for the consultation was satisfactory (i.e The time interval between making contact with the service and appointment for consultation)	4.14 ± 0.94	84.4 (43)	7.8 (4)	7.8 (4)
2. Sufficient explanation was provided regarding the process	4.18 ± 0.71	86.2 (44)	11.8 (6)	2.0 (1)
3. My privacy and confidentiality was respected	4.41 ± 0.61	98.0 (50)	0 (0)	2.0 (1)
4. I was able to see clearly	4.24 ± 0.79	92.2 (47)	3.9 (2)	3.9 (2)
5. I was able to hear clearly	4.00 ± 1.00	80.4 (41)	7.8 (4)	11.8 (6)
6. I was able to express myself adequately using this mode of assessment	4.20 ± 0.78	86.3 (44)	9.8 (5)	3.9 (2)
7. I felt comfortable discussing my problems using this mode of assessment	4.22 ± 0.78	90.2 (46)	3.9 (2)	5.9 (3)
8. The assessment addressed my needs	3.90 ± 0.83	68.6 (35)	27.5 (14)	3.9 (2)
9. The recommendations made were useful	3.88 ± 0.77	72.5 (37)	23.5 (12)	3.9 (2)
10. I am satisfied with the consultation	4.14 ± 0.75	86.3 (44)	9.8 (5)	3.9 (2)
11. I would prefer to use it again?	4.02 ± 0.81	76.5 (39)	19.6 (10)	3.9 (2)
12. I would recommend it to others?	4.08 ± 0.85	86.3 (44)	7.8 (4)	5.9 (3)

Likert Scale: 1-strongly disagree, 2- disagree, 3-neither agree nor disagree, 4- agree, 5- strongly agree

^a^Satisfied: agree or strongly agree, Dissatisfied: disagree or strongly disagree, Unsure: Neither agree nor disagree

A subgroup analysis of the patient feedback scores using Mann Whitney U test (Table 4**)** did not show statistically significant differences in satisfaction on any of the questions based on gender, living situation or prior exposure to our telepsychiatry service. However, satisfaction among outpatients was significantly greater than inpatients on question 1 i.e. ‘The waiting period (time between making contact with the service and appointment for consultation) was satisfactory’ (U = 163.0, *p* = 0.028) and question 4 i.e. ‘I was able to see clearly’ (U = 160.5, *p* = 0.019).

**Table 4:** Differences in patient feedback scores based on their previous contact with the service, gender, living situation and inpatient or outpatient status at the time of the assessment

Questions^a^	Prior telepsychiatry contact with the service			Gender			Living Situation			Status at the time of the assessment		
	Yes = 23 Mean, SD	No = 28 Mean, SD	U, 2 tailed *P* value	Male= 17 Mean, SD	Female = 34 Mean, SD	U, 2 tailed *P* value	Independent living = 41 Mean, SD	Nursing Home = 10 Mean, SD	U, 2 tailed *P* value	Inpatient = 14 Mean, SD	Outpatient = 37 Mean, SD	U, 2 tailed *P* value
Q 1	4.30, 0.765	4.0, 1.054	275.5, 0.339	4.24, 0.831	4.09, 0.996	271.0, 0.696	4.15, 1.014	4.10, 0.568	174.5, 0.432	3.64, 1.324	4.32, 0.784	163.0, 0.028*
Q 2	4.30, 0.765	4.07, 0.663	253.5, 0.150	4.12, 0.781	4.21, 0.687	270.0, 0.674	4.22, 0.759	4.00, 0.471	160.5, 0.242	4.07, 0.616	4.22, 0.750	222.0, 0.386
Q 3	4.43, 0.507	4.39, 0.685	319.0, 0.948	4.41, 0.507	4.41, 0.657	277.0, 0.783	4.41, 0.631	4.40, 0.516	195.0, 0.786	4.36, 0.497	4.43, 0.647	230.0, 0.483
Q 4	4.26, 0.689	4.21, 0.876	321.0, 0.983	4.06, 0.966	4.32, 0.684	246.5, 0.337	4.20, 0.843	4.40, 0.516	186.0, 0.612	3.71, 1.139	4.43, 0.502	160.5, 0.019*
Q 5	3.83, 1.072	4.14, 0.932	269.0, 0.279	4.24, 0.664	3.88, 1.122	253.5, 0.444	4.07, 0.985	3.70, 1.059	160.0, 0.249	3.93, 1.141	4.03, 0.957	252.0, 0.873
Q 6	4.17, 0.937	4.21, 0.630	307.0, 0.755	4.29, 0.588	4.15, 0.857	274.0 0.742	4.27, 0.708	3.90, 0.994	162.5, 0.268	3.93, 0.829	4.30, 0.740	192.5, 0.123
Q 7	4.26, 0.752	4.18, 0.819	307.5, 0.759	4.18, 0.728	4.24, 0.819	268.0 0.639	4.22, 0.822	4.20, 0.632	189.5, 0.681	3.86, 0.949	4.35, 0.676	181.5, 0.067
Q 8	3.87, 0.869	3.93, 0.813	309.5, 0.801	3.88, 0.781	3.91, 0.866	276.5 0.790	3.93, 0.848	3.80, 0.789	183.0, 0.579	3.86, 0.770	3.92, 0.862	242.5, 0.711
Q 9	4.00, 0.798	3.79, 0.738	269.5, 0.276	3.82, 0.728	3.91, 0.793	261.5 0.547	3.90, 0.800	3.80, 0.632	185.0, 0.603	4.00, 0.679	3.84, 0.800	234.0, 0.563
Q 10	4.13, 0.757	4.14, 0.756	319.5, 0.958	4.24, 0.562	4.09, 0.830	272.0 0.705	4.17, 0.771	4.00, 0.667	171.0, 0.368	4.07, 0.616	4.16, 0.800	229.0, 0.480
Q 11	4.17, 0.650	3.89, 0.916	271.0, 0.299	4.00, 0.707	4.03, 0.870	273.5 0.739	4.05, 0.835	3.90, 0.738	178.0, 0.491	3.71, 0.681	4.14, 0.787	186.0, 0.097
Q 12	4.22, 0.600	3.96, 0.999	293.0, 0.537	4.00, 0.935	4.12, 0.808	271.5 0.694	4.10, 0.889	4.00, 0.667	176.0, 0.439	3.86, 0.770	4.16, 0.866	194.5, 0.126

*2 tailed *p* value significance < 0.05; U: Mann Whitney U test statistics

^a^Feedback Questions: Q1:The waiting period for the consultation was satisfactory, Q2: Sufficient explanation was provided regarding the process,Q3: My privacy and confidentiality was respected, Q4: I was able to see clearly, Q5: I was able to hear clearly, Q6:I was able to express myself adequately using this mode of assessment, Q7: I felt comfortable discussing my problems using this mode of assessment, Q8: The assessment addressed my needs, Q9: The recommendations made were useful, Q10: I am satisfied with the consultation, Q11: I would prefer to use it again, Q12.I would recommend it to others

#### Feedback data from clinician’s/ nurses

As shown in Table 5; the mean score of the feedback from clinicians/nurses (*N* = 59, response rate 57.8%) ranged from 4.36–4.73 on all of the 11 questions with over 90% satisfaction. The lowest rates of satisfaction (88%) with a mean score of 4.36 ± 0.78 was for “process of referral was convenient”.

**Table 5:** Feedback for tele-psychiatry consults in 2012 from clinicians/nurses and psychiatrists

Clinicians/Nurses (N = 59)					Psychiatrist (N = 80)				
Feedback Question	Mean ± SD	Satisfied ^a^% (n)	Unsure^b^ % (n)	Dissatisfied^c^ % (n)	Feedback Question	Mean ± SD	Satisfied^a^ % (n)	Unsure^b^ % (n)	Dissatisfied^c^ (n)
The waiting period of the consultation was satisfactory (ie, from the time of referral to consultation)	4.53 ± 0.68	93.2 (55)	5.1 (3)	1.7 (1)	I was able to fit the referral in my existing time schedule	4.13 ± 0.88	86.7 (72)	3.6 (3)	9.6 (8)
The process of referral was convenient	4.36 ± 0.78	88.1 (52)	8.5 (5)	3.4 (2)	The referral had adequate information	4.19 ± 0.96	83.1 (69)	8.4 (7)	8.4 (7)
Adequate privacy and confidentiality was provided during the consultation	4.58 ± 0.91	93.2 (55)	1.7 (1)	5.1 (3)	Adequate privacy and confidentiality was provided	4.08 ± 0.94	81.9 (68)	7.2 (6)	10.8 (9)
The video was clear	4.56 ± 0.90	93.2 (55)	1.7 (1)	5.1 (3)	The video was clear	4.24 ± 1.07	85.5 (71)	3.6 (3)	10.8 (9)
The audio was clear	4.71 ± 0.53	96.6 (57)	3.4 (2)	0 (0)	The audio was clear	4.25 ± 1.03	72 (86.7)	3.6 (3)	9.6 (8)
I felt comfortable participating in the Telemed assessment process	4.68 ± 0.54	96.6 (57)	3.4 (2)	0 (0)	I felt comfortable participating in the Tele-med assessment process	3.78 ± 1.12	66.2 (54)	16.9 (14)	16.9 (14)
The assessment confirmed to our desired outcomes as mentioned on the referral	4.54 ± 0.65	91.5 (54)	8.5 (5)	0 (0)	The time was sufficient to do the assessment and make recommendation	4.36 ± 0.77	94.0 (78)	3.6 (3)	2.4 (2)
The recommendation for management was useful	4.51 ± .65	91.5 (54)	8.5 (5)	0 (0)	The recommendations can be followed up in the community	4.04 ± 0.99	79.5 (66)	10.8 (9)	9.6 (8)
I am satisfied with the consultation	4.63 ± 0.52	98.3 (58)	1.7 (1)	0 (0)	It was a useful adjunct to my routine work	4.25 ± 0.88	85.6 (71)	10.8 (9)	3.6 (3)
I would use it again	4.71 ± 0.46	100 (59)	0 (0)	0 (0)	I preferred it to face to face consultation	3.67 ± 1.39	55.4 (46)	15.7 (13)	28.9 (24)
I would recommend it to others	4.73 ± 0.45	100 (59)	0 (0)	0 (0)	I Would encourage its use	4.45 ± 0.80	92.7 (77)	4.8 (4)	2.4 (2)

Likert Scale: 1-strongly disagree, 2- disagree, 3-neither agree nor disagree, 4- agree, 5- strongly agree

^a^Satisfied: agree or strongly agree (1,2), ^c^Dissatisfied: disagree or strongly disagree (4,5), ^b^Unsure: Neither agree nor disagree (3)

#### Feedback from the psychiatrists/sub-specialty trainee

Table 5 describes the psychiatrist feedback (*N* = 80, response rate: 81.3%). Over the study period, 4 psychiatrists and 1 sub-specialty geriatric psychiatry trainee performed consults. The mean scores on the feedback ranged between 3.67–4.45. The highest mean score was 4.45 ± 0.8 for “I would encourage its use”. The lowest mean score, 3.67 ± 1.39, was for “I prefer it to face to face consultation” but in 46 (55.4%) consults, they agreed with the statement. In 14 (16.9%) consults, psychiatrists did not feel comfortable participating in the assessment.

#### Feedback comments

In the free text comment section of the feedback form, we received 32 comments from patients/significant others, 23 from clinicians/nurses and 38 from psychiatrists. The themes and content are detailed in Table 6**.**

**Table 6:** Themes from feedback comments by consumers, clinicians/referrers and specialists for tele-psychiatry consults in 2012

Themes	Patients		Nurses/Clinicians		Psychiatrist	
	Positive	Negative	Positive	Negative	Positive	Negative
Referral process/information	0	0	Referral process (2)	Referral information requirements (3)	0	Lack of adequate information (2); Difficulty accommodating the referral (2)
Privacy (not sound proof/external noise/intrusions)	0	0	0	Background noise (2)	0	Background noise (5); Intrusions (1)
Audio-visual	0	Audio visual issues (5)	0	Audio-visual issues (5)	0	Technical issues- audio-visual, (5)
Comfort	Comfortable (3)	Daunting (1)	0	0	0	Cold/uncomfortable (3)
Recommendations	Useful recommendations (1)	0	0	0	0	Deficient community resources (1)
Global	Good/Helpful (14)	Unsure (1)	Helpful (4)	0	Useful (4); Suitable (2)	0
Others	Saved time travelling (4); Staff helpful (2); Treated respectfully (1)	0	Educational (2); Supportive (2); Saved time (1)	Interruption by a Medical emergency (2)	Time saving (3)	Patient related issues-behaviour/cognition/hearing (10)

Positive themes from patients described the service as useful and time-saving (e.g. *“we are happy to use it as it saves us a lot of time and traveling”, “very very helpful”, “fantastic)”*. They mentioned that they were treated respectfully (e.g. *“the people were good and understanding”*). Negative themes were predominantly related to audio-visual difficulties (e.g. *“TV (visual) not working”, “forgot my hearing aids and eyes were blurry”, “couldn’t hear clearly”)*. Mixed comments had elements of uncertainty (e.g. *“Unsure, but every effort is being made to help me”*).

Positive themes from clinicians made references to telepsychiatry as helpful, educational and easy to access (e.g. *“fantastic service for rural and remote people”, “great to have a discussion/ educational opportunity at the end”).* They also valued the support and the time saved (e.g. *“thank you for support and advice”, “Telepsychiatry has meant a greatly reduced waiting time for this consult”).* Some clinicians expressed concerns regarding the information required for the referral (e.g. *“the referral process took a while as I had to wait for the GP and then had to include the other assessment tools”)* and audio-visual issues*, “visual not working”, “I had not anticipated the hearing problem for the client. Repeating the questions was awkward and made the consultation difficult for both parties”).*

Psychiatrists also found telepsychiatry to be useful and time-saving for rural patients (e.g. *“was very useful to do telemed consultation in this patient- a lot quicker”).* Negative themes mentioned difficulties due to patient-related issues such as disabilities and poor engagement (*eg. “Advanced dementia precluded the assessment but it was still helpful”, “patient was not engaging and so the assessment was difficult”)* or technical aspects like audio-visual difficulties, noise, cold and privacy issues (e.g. *“visual not clear”, “too cold in the room- uncomfortable”, “there was a lot of background noise at the far end”*).

## Discussion

This study focuses on the clinical role of telepsychiatry for older adults, when embedded within a larger psychiatry service. It is a crucial adjunct to the visiting service in the region which is infrequent and cost intensive, often unable to cater for all needs of the community [12]. It is also the only service evaluated which caters to older adults resident in the community, nursing homes and those admitted in community hospitals providing a unique opportunity to compare satisfaction in different settings within the rural and remote community.

### Utilization patterns

The pattern of utilization shows that in remote areas, where psychiatry services may not be readily accessible, a community-based telepsychiatry program is applicable in a broad range of psychiatric disorders from adjustment disorders to severe mental illnesses apart from dementia. This is in contrast to most other telepsychiatry programs evaluated among older adults, where the most common diagnosis is dementia [6, 7]. Moreover, a high proportion of patients (71.6%) also had 2 or more medical conditions, emphasizing the need for a specialist input in assessment and management of psychiatric disorders. Thus, telepsychiatry can be used to bridge the gap in specialist psychiatry services for older adults resident in rural and remote communities, not only for dementia, but also for other psychiatric disorders.

Telepsychiatry can be a useful means of providing a cohesive management plan with the psychiatrist input as evident by the recommendations for psychosocial interventions. On the other hand, it also allowed evaluation of patients admitted in community general hospitals and severely ill patients in the community who needed urgent intervention such as specialist inpatient transfer or further medical evaluation as evident in the recommendations made (Table 1).

Majority of the patients reside independently in the community, similar to the rates in a psychogeriatric outpatient clinic where only about 14% were in nursing homes [13]. Telepsychiatry in nursing homes is feasible and acceptable [6, 14], however, compared to patients living independently in the community, its use by patients in nursing homes was lower. A community based program expectedly has a different pattern of utilization but one of the factors possibly hampering its use by the nursing home population in this program was the lack of mobile telepsychiatry set-up within the nursing homes linked to the secure video-conferencing system. Patient disabilities make transport to the nearest facility with a secure network challenging. A qualitative evaluation of satisfaction with mental health service delivery among patients with dementia and their care givers found that services within the home setting were less distressing and more empowering than being transported to a clinic which is not only onerous but also worsens the confusion and distress [15]. To improve the reach of a community-based service in nursing homes, or even among the more disabled population in the community, it may help to use compatible mobile video-conferencing units which bring the equipment to their place of residence than having to transport them. Another factor could be the limitation of its use in patients with sensory or cognitive disabilities common in the nursing home population, a point raised by all stakeholders in their feedback comments. It may help to support the assessment by hearing and visual aids in the presence of trained staff to facilitate the process. This would help to improve the access and overcome the challenges to its use among older adults with cognitive or sensory disabilities. Another option for the more disabled populations is to focus the visiting service on this population and use telepsychiatry for urgent support. This can help with efficient use of resources and maximize the access.

Acutely unwell patients with difficult behaviors formed a small percentage (31.3%) of the assessments (Table 1), similar to another community-based program [7]. Telepsychiatry may not be appropriate for violent, unstable, or impulsive individuals [16], though the most recent guidelines [17] do not discourage its use in acutely disturbed patients. In their feedback comments, psychiatrists mentioned the difficulty engaging with patients with behavioral symptoms or cognitive difficulties (Table 6). The concerns in a in-person consult may be similar where it becomes important to assess non-verbal behaviors and obtain collateral information. In telepsychiatry, these barriers can be overcome by involving the clinicians and patients’ family along with improved visual and audio quality.

### Feedback on acceptance and satisfaction

Though the population sample used for evaluating the satisfaction was different from the one used for the utilization pattern, the two samples were very similar in their mean age, gender distribution, as well as their primary psychiatric diagnoses. Thus, even though two different samples were used, the two findings tie in together to tell a coherent story.

#### Patient satisfaction

Mean scores ranging from 3.88–4.41 is suggestive of comparable acceptability to other population groups and specialties [18–20]. The overall satisfaction with the consultation was highly rated. In case of severe dementia, delirium and severe psychotic illness, we received feedback from 5 family members which provides some input on behalf of the patients. Eventhough overall satisfcation was high, patients admitted in community hospitals at the time of telepsychiatry assessment were significantly less satisfied with wait times and visual clarity compared to outpatients and this could be because of subjective differences in needs/expectations or illness severity. A study on an acute inpatient unit showed lower satisfaction across all domains in patients with a psychotic illness compared to those without psychosis [21]. Since inpatient admissions are primarily to safely manage difficult behaviors and acute disturbances, some of which may be psychotic symptoms, this difference may not be unusual. A comparison of inpatient and outpatient community setting has never been done and may need further evaluation.

The feedback response rate among patients (52%) is lower than most other studies (60%) [20]. The feedback response rate was significantly low among inpatients in community hospitals. We postulate that this was possibly because of illness severity and non-availability of a family member as these consults are done as a priority, which may not provide sufficient time to engage the family. Also, administratively, it was challenging to make the feedback forms available in a timely manner given the multiple sites. Our study did not have an alternative if the feedback forms were missed at the end of the consult, for example, if the patient left before providing the feedback.

#### Satisfaction among clinicians/nurses

The mean score of 4.36–4.74 is similar to some other studies [7, 22]. Telepsychiatry appears to be a well-accepted mode of assisting our clinicians/nurses in their clinical work. As reflected in their comments, telepsychiatry was seen as an important support and an opportunity for enhancing clinical skills (Table 6). Some expressed their displeasure with the referral process, needing much information and referral signed by the GP, which is similar to the experience of the community program at Baycrest [7].

#### Satisfaction among psychiatrists

Our findings on psychiatrist satisfaction feedback were similar to another study where they were keen to encourage its use in older adults but in some scenarios, they expressed doubts regarding telepsychiatry over an in-person consultation [23] .

Psychiatrists expressed a high level of satisfaction with the technical aspects, time available and feasibility of the recommendations in the community. Some of their comments revealed concerns regarding the physical environment (e.g. noise and temperature) and lack of comfort in situations where cognitive or sensory disabilities and behavioural symptoms were prominent (Table 6). This is an important aspect to consider in resource allocation and structuring of services. In severely ill or disabled older adults, telepsychiatry can be used for urgent review and support, but more detailed assessment could be the focus of the visiting service. Given that all psychiatrists were from the same service and there was only one trainee, any comparison within the group was not done.

#### Technical challenges

The dissatisfaction with the audio among patients (6, 11%) was higher than what is seen among younger adults (less than 5%) [24, 25]. Means scores for satisfaction with the audio were lower among patients compared to other stakeholders (psychiatrists and clinicians/nurses, Tables 3 & 5). Both these observations reflect possible sensorineural deficits among older adults. The psychiatrists and clinicians expressed a sense of discomfort when patients had hearing difficulties. Use of hearing devices or noise cancellation could improve the satisfaction but has never been assessed. Some of the challenges can be overcome by involving the family and clinicians in the process as observed in another study in a memory clinic [18]. This is a crucial aspect for application of telepsychiatry in this population.

This evaluation was carried out a few year ago and while the data may be old, not much has changed over the years. There have been no further studies published in the field of community based telepsychiatry service models for the elderly. The service under evaluation continues to use the hub and spoke model with the use of telepsychiatry as an adjunct to the visiting service. Data derived from this evaluation has helped the service review its processes and resource allocation. Listening devices were introduced but were perceived to be complex and often not used. With advancing technology, it may be possible to develop better listening devices which could be used with ease. Usability and usefulness of the equipment has been identified as one of the important barriers in adoption of technology in one qualitative study which in turn stresses on the need for innovation [26]. Synchronizing mobile devices to a secure network such that the video and audio quality remains reliable has been a challenge. This is especially so in terms of meeting the confidentiality and privacy requirements of the health system. Given the challenges of incorporating technology in day to day mental health service delivery, especially for elderly with significant cognitive and sensory deficits, the service has reorganized its visiting service as a means of assessment of more disabled population in the community and nursing homes, while telepsychiatry continues to be used for specialist input in cases of less disabled population and for urgent support to the clinicians and nursing home staff. Over the past few years, the use of telemedicine as a means of education and support to the staff in rural and remote has gained momentum through the Extended Community Health Outcomes (ECHO) platform [27] and the current service uses videoconferencing as a means of education and support to the clinicians in the community, though not as part of the formal ECHO platform. Thus, the findings and recommendations remain equally relevant and raise important questions about future direction of community based telepsychiatry services for elderly.

### Strengths and limitations

Our study is the only one in Australia evaluating a community-based telepsychiatry program for older adults and only second to the one evaluated at Baycrest, Toronto. The greater area of coverage and population with diverse diagnoses, besides dementia, represents the strength of a community-based service.

However, there are various limitations. Firstly, this is a descriptive study. Secondly, the time frame for feedback evaluation is brief, but the large numbers of consults, make it comparable to other studies. Thirdly, being a single Centre study, the findings may be limited to the service under evaluation and should be generalized with care. Fourthly, the feedback tools used were not standardized or validated. They were adapted from other telepsychiatry surveys and reviewed internally. This was done as there are no standardized feedback surveys in telepsychiatry that could be used across various stakeholders. Fifthly, the results regarding satisfaction were limited by a low response rate from patients and clinicians. This raises questions about the representativeness of our sample. Comparison of the survey responders and non-responders among the patient group did not reveal any significant differences except lower feedback from in-patients. Future studies should incorporate alternative strategies such as mailing the feedback if it is missed or follow up over the phone so as to maximize the feedback. We also do not have a follow up of the telepsychiatry consults to measure the outcomes. Never the less, it does provide a direction for future studies in this area.

## Conclusions

In rural and remote areas:

1. A community-based telepsychiatry program can be useful for assessment and input in a broad range of psychiatric disorders among older adults.
2. It is well accepted by various stakeholders and can be a useful means of supporting the community services.
3. Satisfaction among patients admitted in hospital was lower than those seen as outpatients.
4. Sensory disabilities and illness related deficits were a major area of dissatisfaction among all stakeholders even with the use of high speed internet and advanced equipment.

### Future directions and recommendations for the use of telepsychiatry among older adults

1. Use of community-based telepsychiatry programs for a broad range of psychiatric disorders can help to close the gap in rural and remote regions and expand the reach of psychiatrist services.
2. Given the limitation with the use of telepsychiatry in patients with disability, telepsychiatry should be used as a means of collaboration with existing community services and support the visiting service to enable efficient use of resources. This seems to concur with previous studies [5] .
3. Further evaluation is needed for difference in satisfaction with the use of telepsychiatry among inpatients compared to outpatients and implicating factors.
4. More robust research is needed on technological innovations specific to tele-psycho-geriatrics such as using portable devices, hearing or visual aids and background noise cancellation strategies.
